# Tobacco Insomnia

**Published:** 1896-01

**Authors:** 


### SELECTIONS.

### Tobacco Insomnia.

Many brain-workers suffer from inability to sleep. This is frequently met with among those who work late at night. The sufferers complain that they feel most lively just when the time for retiring has come, and that a long period of restlessness precedes a troubled slumber, from which the slightest noise awakens them. This is very often caused almost entirely by an over-indulgence in tobacco. They smoke just before going to bed, ignorant of the fact that not only may tobacco prevent sleep temporarily, but it may render it less deep, and consequently less refreshing. A grave responsibility attaches to those who lightly seek to relieve a symptom which is really a warning by recourse to a dangerous palliative. The inability to sleep is often merely the outcome of an unnatural mode of life, and if this be corrected the disability disappears of itself. Men who work late are commonly addicted to the tobacco habit. To them tobacco is not a relaxation after a day's work, but a nerve stimulant which enables them to accomplish tasks which would otherwise be difficult of accomplishment. When the mouth becomes dry, alcohol in some form or other is resorted to, as a fillip to enable the smoker to tolerate still another cigar or two. Under these circumstances tobacco acts as a cerebral irritant and interferes with the vaso-motor centres of the brain to such an extent that the vessels are unable to adjust themselves forthwith to the condition required for healthy and untroubled sleep. Discretion in tobacco use would save many from this distressing condition of chronic insomnia. Smoking early in the day should be discountenanced, and it is equally undesirable within an hour or so of retiring to rest. The best remedy for the tobacco habit, short of total abstention, is to take a short walk in the open air after the last pipe. Under no circumstances should drugs be used for this form of nocturnal restlessness.—*Pacific Medical Journal.*

